# Prevalence and clinical implications of bronchiectasis in patients with overlapping asthma and chronic rhinosinusitis: a single-center prospective study

**DOI:** 10.1186/s12890-021-01575-7

**Published:** 2021-07-05

**Authors:** Haiyan Sheng, Xiujuan Yao, Xiangdong Wang, Yuhong Wang, Xiaofang Liu, Luo Zhang

**Affiliations:** 1 Department of Respiratory and Critical Care Medicine, Beijing Tongren Hospital, Capital Medical University, No. 1, Dongjiao Minxiang, Dongcheng District, Beijing, 100730 China; 2 Department of Otolaryngology Head and Neck Surgery, Beijing Tongren Hospital, Capital Medical University, Beijing, China; 3 Key Laboratory of Otolaryngology Head and Neck Surgery of Ministry of Education of China, Beijing Institute of Otolaryngology, No. 17, Hougou Hutong, Dongcheng District, Beijing, 100005 China

**Keywords:** Asthma, Bronchiectasis, Chronic rhinosinusitis, Nasal polyps

## Abstract

**Background:**

As a typical “united airway” disease, asthma-chronic rhinosinusitis (CRS) overlap has recently drawn more attention. Bronchiectasis is a heterogeneous disease related to a variety of diseases. Whether bronchiectasis exists and correlates with asthma-CRS patients has not been fully elucidated. The purpose of the study was to explore the presence and characteristics of bronchiectasis in patients with overlapping asthma and CRS.

**Methods:**

This report describes a prospective study with consecutive asthma-CRS patients. The diagnosis and severity of bronchiectasis were obtained by thorax high-resolution computed tomography (HRCT), the Smith radiology scale and the Bhalla scoring system. CRS severity was evaluated by paranasal sinus CT and the Lund-Mackay (LM) scoring system. The correlations between bronchiectasis and clinical data, fraction of exhaled nitric oxide, peripheral blood eosinophil counts and lung function were analyzed.

**Results:**

Seventy-two (40.91%) of 176 asthma-CRS patients were diagnosed with bronchiectasis. Asthma-CRS patients with overlapping bronchiectasis had a higher incidence rate of nasal polyps (NPs) (*P* = 0.004), higher LM scores (*P* = 0.044), higher proportion of ≥ 1 severe exacerbation of asthma in the last 12 months (*P* = 0.003), lower postbronchodilator forced expiratory volume in one second (FEV_1_) % predicted (*P* = 0.006), and elevated peripheral blood eosinophil counts (*P* = 0.022). Smith and Bhalla scores were shown to correlate positively with NPs and negatively with FEV_1_% predicted and body mass index. Cutoff values of FEV_1_% predicted ≤ 71.40%, peripheral blood eosinophil counts > 0.60 × 10^9^/L, presence of NPs, and ≥ 1 severe exacerbation of asthma in the last 12 months were shown to differentiate bronchiectasis in asthma-CRS patients.

**Conclusions:**

Bronchiectasis commonly overlaps in asthma-CRS patients. The coexistence of bronchiectasis predicts a more severe disease subset in terms of asthma and CRS. We suggest that asthma-CRS patients with NPs, severe airflow obstruction, eosinophilic inflammation, and poor asthma control should receive HRCT for the early diagnosis of bronchiectasis.

## Background

The “united airways” concept indicates that upper and lower airway diseases often coexist and share similar pathogenic mechanisms, and asthma-chronic rhinosinusitis (CRS) overlap is a typical “united airways” disease [1–3]. It was shown that CRS was associated with more severe asthma, especially in CRS with nasal polyp (NP) patients [2, 4].

Bronchiectasis is a heterogeneous disease characterized by permanent and irreversible destruction and dilatation of the bronchial wall induced by chronic airway inflammation [5]. The overlap of asthma and bronchiectasis in the same patients has been described in several observations [6, 7]. Asthma can be diagnosed in approximately 50% of patients with bronchiectasis [8, 9]; in turn, bronchiectasis has been diagnosed in 3–47% of patients with asthma [6, 10]. The coexistence of asthma and bronchiectasis may indicate a potentially specific disease phenotype with distinct clinical features and therapeutic options [11]. The correlation between CRS with NPs (CRSwNPs) and bronchiectasis has also been reported [12]. The prevalence of CRS in patients with bronchiectasis is 45–77% [3, 12–14]. Bronchiectasis patients with CRS are shown to have significantly more exacerbations and worse quality of life than bronchiectasis patients without CRS involvement [14], indicating that the coexistence of CRS in bronchiectasis patients represents a more severe disease subset.

Traditionally, bronchiectasis is characterized by neutrophilic inflammation and closely linked to persistent bacterial infection [5]. Nevertheless, the inflammatory characteristics of bronchiectasis in asthma or CRS were controversial in previous studies. Some records indicated that bronchiectasis presented eosinophilic inflammation in asthma or CRS patients [15, 16], while others drew the opposite conclusion [7, 17]. Given these discrepancies, it is therefore necessary to investigate the inflammatory characteristics of bronchiectasis in asthma-CRS patients.

Although there have been several studies involving the association of bronchiectasis with asthma or CRS, to date, whether bronchiectasis exists and correlates with asthma-CRS patients has not been fully elucidated. In this study, we aimed to assess the prevalence, inflammatory characteristics, and clinical implications of bronchiectasis in a prospective cohort of asthma-CRS patients.

## Methods

### Study and participants

This report describes a prospective study. Outpatients diagnosed with asthma-CRS were consecutively enrolled at the Department of Respiratory Medicine and Otorhinolaryngology of Beijing Tongren Hospital from Jan 2016 to Dec 2019. Asthma diagnosis was made according to the Global Initiative for Asthma (GINA) criteria [18]: (1) a definite clinical history of asthmatic symptoms; and (2) hyperbronchodilator reversibility with an increase in forced expiratory volume in one second (FEV_1_) > 12% and > 200 mL from baseline and/or airway hyperresponsiveness with a decrease in FEV_1_ from baseline of ≥ 20% with standard doses of methacholine. All participants were stable for at least 1 month without oral corticosteroids. Severe asthma was defined as an uncontrolled condition despite step 4 or 5 therapy and treatment of contributory factors or worsened when high-dose treatment was decreased according to the GINA criteria [18]. Severe exacerbation of asthma was defined as an asthma attack that needed emergency department attendance, hospitalization, or the need for oral corticosteroids [18]. CRS was diagnosed by the guidelines of the European Position Paper on Rhinosinusitis and Nasal Polyps 2020 [3]. Patients were excluded if any of the following applied: (1) diagnosis of bronchiectasis prior to asthma; (2) chronic obstructive pulmonary disease (COPD) or any other significant respiratory diseases; (3) pneumonia or measles in childhood, primary ciliary dysfunction, tuberculosis or other diseases that can interfere with bronchiectasis; (4) pregnancy; (5) cancer; (6) severe heart failure; and (7) smoking index > 20 pack-years.

Clinical data were collected, while peripheral blood eosinophil counts, serum immunoglobulin E (IgE) levels, fraction of exhaled nitric oxide (FeNO), lung function, paranasal sinus CT and thorax high-resolution CT (HRCT) were examined and analyzed.

This study complied with the Declaration of Helsinki and was approved by the Ethics Committee of Beijing Tongren Hospital, Capital Medical University (approval number: TRECKY2013-KS-37). Written informed consent was obtained from all recruited patients.

### FeNO analysis

Patients were instructed to exhale at a flow rate of 50 mL/s through a disposable mouthpiece by the NIOX electrochemical analyzer (Aerocrine, Sweden) [19]. Measurements were performed at least three times, and the average was calculated for analysis.

### Asthma assessment with spirometry

Postbronchodilator FEV_1_ and forced vital capacity (FVC) were measured by a spirometer (Jaeger, Germany). The procedure was performed in accordance with current ATS/ERS guidelines [20].

### Radiological diagnosis and severity assessment of bronchiectasis by using HRCT

Chest HRCT (Phillips Company, Amsterdam, the Netherlands) images were obtained in full inspiration with 1-mm collimation. A broncho-arterial ratio > 1 in HRCT images was diagnosed as bronchiectasis radiologically [21]. Smith scores were used to estimate the extent of bronchiectasis in each lobe [22]: 0 if there was no evidence of bronchiectasis, and 1–4 if < 25%, 25–49%, 50–74% and ≥ 75% of the bronchi were abnormal, respectively. The lingula was graded as a separate lobe, and the maximum possible score was 24. Patients with Smith scores ≥ 3 were assigned to the bronchiectasis group as previously described [23]. Meanwhile, the Bhalla scoring system was used to estimate the severity of bronchiectasis in each lobe [24]: 0 if no involvement, and 1–3 if the broncho-arterial ratio was < 2, 2–3, and > 3, respectively. Two thoracic radiologists ranked the Smith and Bhalla scores independently, and the final score of each lobe was the average.

### CRS analysis using paranasal sinus CT

Patients received paranasal sinus CT examination (Phillips Company), the LM staging system was applied to rank the severity of sinusitis, and the average was scored by two independent radiologists blinded to the clinical status [25]. The sinus groups included the maxillary, frontal, sphenoidal, anterior ethmoidal, and posterior ethmoidal sinuses. Each sinus group was subsequently assigned a numeric grade: 0, if no abnormality; 1, if partial opacification; and 2, if total opacification. The condition of the ostiomeatal complex was simply scored as 0 if not obstructed or 2 if obstructed. The total score range was from 0 to 24 [25].

### Statistical analysis

The mean ± standard deviation (SD) with 95% confidence interval (CI), median (interquartile range) and frequencies/percentages were used to show parametric, nonparametric and categorical variables, respectively. The unpaired t test or Mann–Whitney U test was used to determine the significance of continuous variables as appropriate. The chi-square test was used to determine the significance of categorical variables. Line regression was used to test the correlation between the severity of bronchiectasis and the clinical parameters. Multivariate logistic regression and multivariate linear regression were used to calculate coefficients or odds ratios as appropriate. Statistical analysis was carried out by using SPSS 21.0 software. A *P* value < 0.05 was considered to be significant.

The receiver operating characteristic (ROC) curve, area under the curve (AUC), and the optimal cutoff value of postbronchodilator FEV_1_% predicted, ≥ 1 severe exacerbation of asthma in the last 12 months, peripheral blood eosinophil counts and postbronchodilator FEV_1_% predicted for bronchiectasis were calculated according to the method described by Hanley and McNeil [26]. As continuous test variables, peripheral blood eosinophil counts and postbronchodilator FEV_1_% predicted were converted to dichotomous state variables based on the ideal cutoff values. Subsequently, dichotomous state variables of peripheral blood eosinophil counts, postbronchodilator FEV_1_% predicted, NP, and ≥ 1 severe exacerbation of asthma in the last 12 months as covariates established a multiple logistic regression and were subsequently conducted to acquire a predictive equation for a combined model. ROC curves were then determined for the 5 dichotomous state variables. Finally, using the 5 dichotomous state variables, differentiation of bronchiectasis was performed by comparing their AUCs using MedCalc software.

## Results

### Patient characteristics

A total of 176 patients (mean age, 53.90 ± 14.26 years) were included in the study and 89 (50.57%) were males. The median asthma duration was 6.50 (2.00, 18.00) years. Fifty-four (30.68%) patients experienced at least one severe exacerbation of asthma in the last 12 months. A total of 82 (46.59%) patients suffered from NPs. Bronchiectasis was present in 72 (40.91%) patients. Patient characteristics are summarized in Table 1.

**Table 1:** Baseline patient characteristics (n = 176)

Characteristics	Values
Male sex, n (%)	89 (50.57)
Age, y	53.90 ± 14.26 (51.78–56.03)
BMI, kg/m^2^	24.56 ± 3.94 (23.98–25.15)
Positive smoking status^a^, n (%)	55 (31.25)
Smoking index^b^, pack-years	10.00 (8.00,14.00)
Duration of asthma, y	6.50 (2.00,18.00)
NPs, n (%)	82 (46.59)
Prior sinus surgery, n (%)	34 (19.32)
Allergic rhinitis, n (%)	112 (63.64)
Atopic dermatitis, n (%)	14 (7.95)
Gastroesophageal reflux disease, n (%)	14 (7.95)
ICS dose (fluticasone equivalent), μg/d	320.00 (160.00, 320.00)
Severe asthma, n (%)	26 (14.77)
≥ 1 severe exacerbation of asthma in the last 12 months, n (%)	54 (30.68)
≥ 1 pneumonia in the last 12 months, n (%)	51 (28.98)
Peripheral blood eosinophil counts, × 10^9^/L	0.37 (0.20,0.70)
FeNO, ppb	36.50 (18.00, 66.00)
Total IgE, IU/mL	177.00 (48.83, 356.50)
Atopy, n (%)	91 (51.70)
Postbronchodilator FEV_1_% predicted, %	79.10 ± 19.91 (76.13–82.06)
LM scores	10.00 (6.00, 17.00)
Bronchiectasis in HRCT, n (%)	72 (40.91)

Parametric data are expressed as the mean ± SD (*95% CI*); nonparametric data are expressed as the median (interquartile range). ICS, inhaled corticosteroid; 2 μg beclomethasone = 2 μg budesonide = 1 μg fluticasone

*BMI* body mass index, *NPs* nasal polyps, *FEV*_*1*_ forced expiratory volume in 1 s, *FeNO* fractional exhaled nitric oxide, *IgE* immunoglobulin E, *LM* Lund-Mackay

^a^Positive smoking status included ex- or current-smokers

^b^Smoking history in patients with positive smoking status

### Characteristics of asthma-CRS patients overlapping with bronchiectasis

**Fig. 1:**
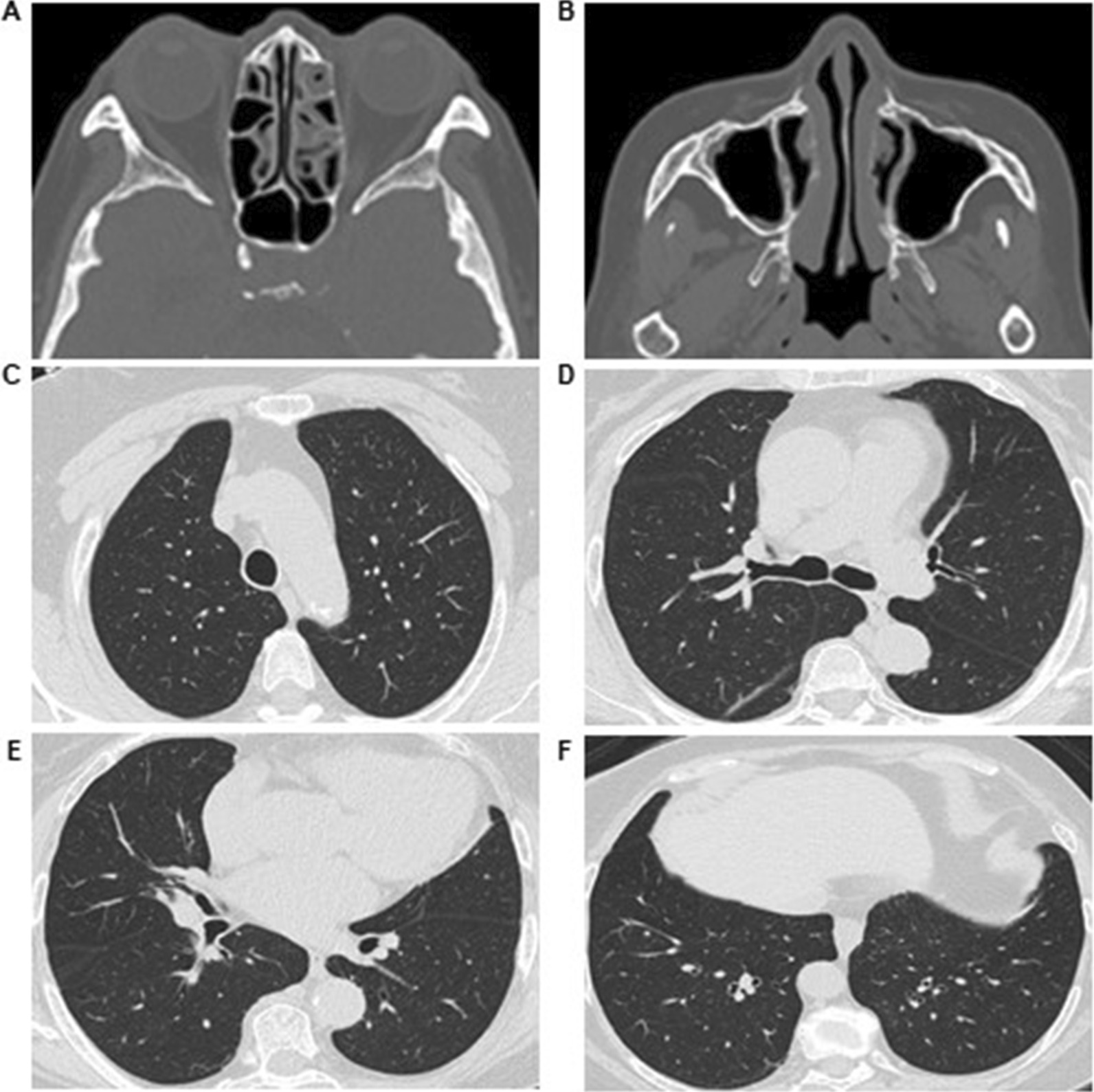
Typical imaging findings of the nonbronchiectasis group. A 65-year-old female suffered from asthma for 3 years and CRS for 1 year. **A**, **B** Paranasal sinus CT imaging revealing CRS involving maxillary and ethmoidal sinuses without the existence of nasal polyps. **C**–**F** Lung windows of HRCT were normal

**Fig. 2:**
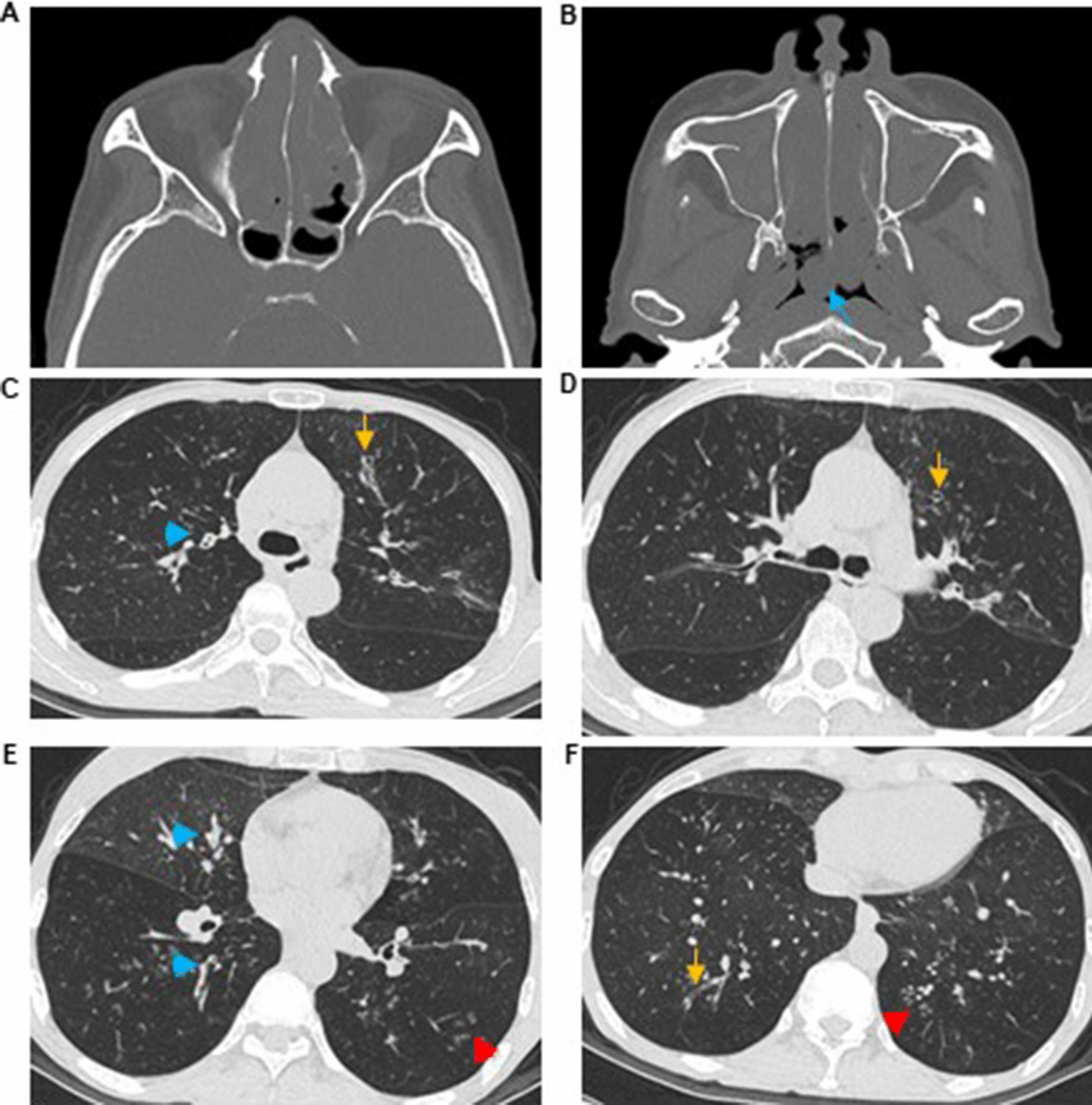
Typical imaging findings of the bronchiectasis group. A 55-year-old female suffered from asthma for 30 years and CRS for 20 years and experienced one severe exacerbation of asthma in the last 12 months. **A**, **B** Paranasal sinus CT imaging revealing CRS involving the whole sinuses with the existence of nasal polyps (blue arrow). **C**–**F** Lung windows of HRCT depicting extensive bronchiectasis (yellow arrow), with thickened bronchial walls (blue arrowhead) and the presence of a tree-in-bud pattern (red arrowhead)

The patients were allocated into 2 different subgroups, asthma-CRS patients with and without bronchiectasis, for further comparison. As shown in Table 2 and Figs. 1 and 2, patients with bronchiectasis showed a higher frequency of severe exacerbation of asthma in the last 12 months (*P* = 0.003), elevated peripheral blood eosinophil counts (*P* = 0.022) and total IgE levels (*P* = 0.044), lower FEV_1_% predicted (*P* = 0.006), higher LM scores (*P* = 0.044) and increased occurrence rates of NPs (*P* = 0.004). There were no observed differences in sex, age, smoking status, sinus surgery history, prevalence of atopic diseases, inhaled corticosteroid (ICS) dose, or ratio of ≥ 1 pneumonia in the last 12 months between the two groups.

**Table 2:** Comparison of characteristics of patients with bronchiectasis versus nonbronchiectasis

Variables	Nonbronchiectasis group (n = 104)	Bronchiectasis group (n = 72)	*P* Value
Male sex, n (%)	51 (49.04)	38 (52.78)	0.626
Age, y	53.28 ± 14.66 (50.43–56.13)	54.81 ± 13.71 (51.58–58.03)	0.487
BMI, kg/m^2^	24.87 ± 3.61 (24.17–25.57)	24.12 ± 4.37 (23.09–25.14)	0.213
Positive smoking status^a^, n (%)	31 (29.81)	24 (33.33)	0.620
Smoking index^b^, pack-years	10.00 (8.00,14.00)	8.00 (6.25,13.50)	0.113
Duration of asthma, y	5.50 (1,12.75)	8.00 (2.00, 20.00)	0.171
NPs, n (%)	35 (33.65)	40 (55.56)	0.004
Prior sinus surgery, n (%)	16 (15.38)	18 (25.00)	0.112
Allergic rhinitis, n (%)	63 (60.58)	49 (68.06)	0.311
Atopic dermatitis, n (%)	11 (10.58)	3 (4.17)	0.122
Gastroesophageal reflux disease, n (%)	9 (8.65)	5 (6.94)	0.680
ICS dose (fluticasone equivalent), μg/d	320.00 (160.00, 320.00)	285.00 (160.00, 320.00)	0.713
Severe asthma, n (%)	11 (10.58)	15 (20.83)	0.059
≥ 1 severe exacerbation of asthma in the last 12 months, n (%)	23 (22.12)	31 (43.06)	0.003
≥ 1 pneumonia in the last 12 months, n (%)	27 (25.96)	24 (33.33)	0.289
Peripheral blood eosinophil counts, × 10^9^/L	0.32 (0.15, 0.58)	0.44 (0.23, 0.90)	0.022
FeNO, ppb	32.00 (18.00, 61.75)	44.00 (23.00,74.75)	0.056
Total IgE, IU/mL	135.50 (48.10, 285.50)	232.00 (56.75,525.25)	0.044
Atopy, n (%)	57 (54.81)	34 (47.22)	0.322
Postbronchodilator FEV_1_% predicted, %	83.09 ± 16.47 (79.89–86.30)	73.32 ± 22.94 (67.93–78.71)	0.006
LM scores	9.00 (6.00, 15.75)	11.50 (7.25, 18.00)	0.044
Smith scores of bronchiectasis		7.56 ± 3.46 (6.75–8.37)	
Bhalla scores of bronchiectasis		3.97 ± 1.50 (3.62–4.32)	

Parametric data are expressed as the mean ± SD (*95% CI*); nonparametric data are expressed as the median (interquartile range)

*BMI* body mass index, *NPs* nasal polyps, *FEV*_*1*_ forced expiratory volume in 1 s, *ICS* inhaled corticosteroid, *FeNO* fractional exhaled nitric oxide, *IgE* immunoglobulin E, *LM* Lund-Mackay

^a^Positive smoking status included ex- and current smokers

^b^Smoking history in patients with positive smoking status

### Coexistence of bronchiectasis correlates with disease severity in asthma-CRS patients

Logistic analysis indicated that the presence of bronchiectasis was positively correlated with NPs (*OR* 2.79; *95% CI* 1.43–5.46), ≥ 1 severe exacerbation of asthma in the last 12 months (*OR* 2.14; *95% CI* 1.02–4.51) and peripheral blood eosinophil counts (*OR* 2.60; *95% CI* 1.19–5.67) and negatively associated with postbronchodilator FEV_1_% predicted (*OR* 0.98; *95% CI* 0.96–1.00) (Table 3).

**Table 3:** Logistic regression analyses for bronchiectasis

Variables	Bronchiectasis: logistic regression		
	*OR*	*95% CI*	*P* value
NPs	2.79	1.43 to 5.46	0.003
≥ 1 severe exacerbation of asthma in the last 12 months	2.14	1.02 to 4.51	0.045
Peripheral blood eosinophil counts	2.60	1.19 to 5.67	0.016
Postbronchodilator FEV_1_% predicted	0.98	0.96 to 1.00	0.039

*FEV*_*1*_ forced expiratory volume in 1 s, *NPs* nasal polyps

**Fig. 3:**
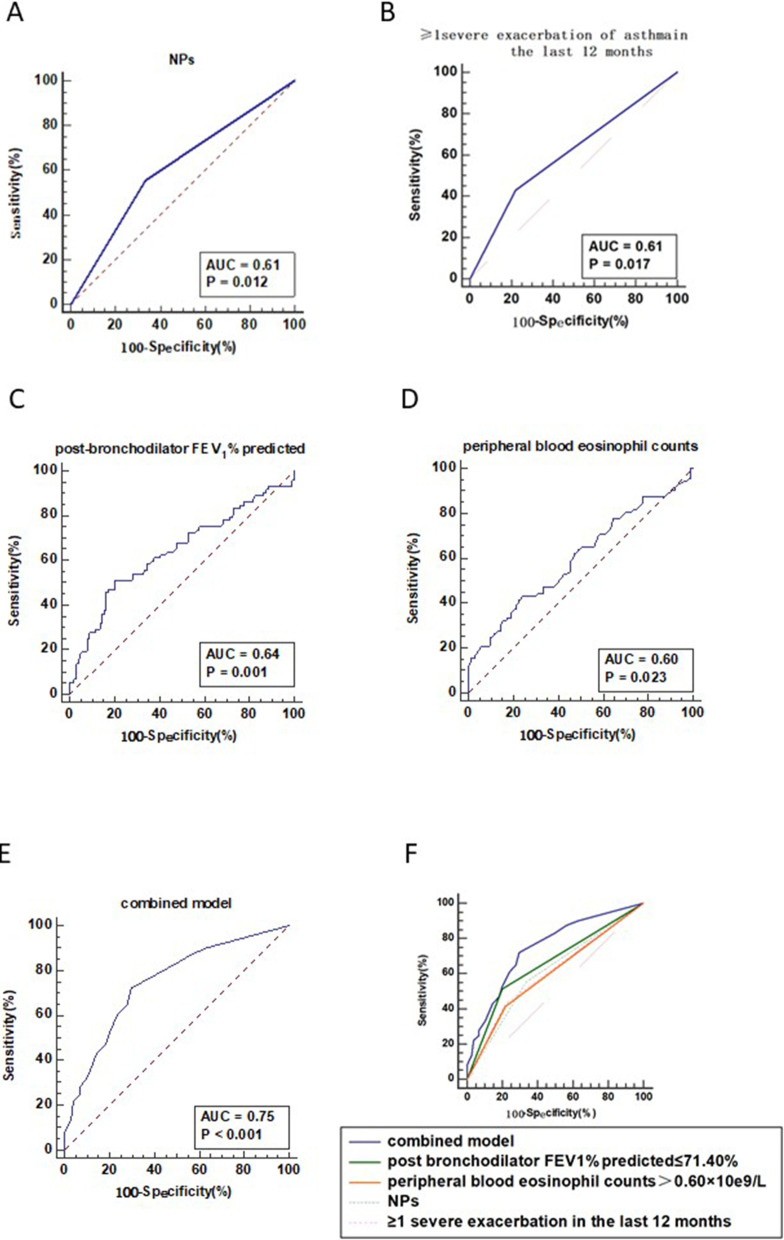
ROC curves to assess the diagnostic accuracy for bronchiectasis in asthma-CRS overlap patients. **A** ROC curve of NPs for assessing the diagnostic accuracy for bronchiectasis. **B** ROC curve of ≥ 1 severe exacerbation of asthma in the last 12 months for assessing the diagnostic accuracy for bronchiectasis. **C** ROC curve of postbronchodilator FEV_1_% predicted for assessing the diagnostic accuracy for bronchiectasis. **D** ROC curve of peripheral blood eosinophil counts for assessing the diagnostic accuracy for bronchiectasis. **E** ROC curve of the combined model with postbronchodilator FEV_1_% predicted ≤ 71.4%, peripheral blood eosinophil counts > 0.6 × 10^9^/L, NPs, and ≥ 1 severe exacerbation of asthma in the last 12 months for assessing the diagnostic accuracy for bronchiectasis. **F** ROC curves of the combined model, postbronchodilator FEV_1_% predicted ≤ 71.4%, peripheral blood eosinophil counts > 0.6 × 10^9^/L, NPs, and ≥ 1 severe exacerbation of asthma in the last 12 months to assess the diagnostic accuracy for bronchiectasis

ROC curves were created to assess the diagnostic accuracy and optimal cutoff values of NPs, ≥ 1 severe exacerbation of asthma in the last 12 months, postbronchodilator FEV_1_% predicted, peripheral blood eosinophil counts, and a combined model for bronchiectasis within the asthma-CRS patients (Fig. 3). The AUC values of NPs, ≥ 1 severe exacerbation of asthma in the last 12 months, postbronchodilator FEV_1_% predicted and peripheral blood eosinophil counts in diagnosing bronchiectasis were 0.61 (*95% CI* 0.53–0.68), 0.61 (*95% CI* 0.53–0.68), 0.64 (*95% CI * 0.57–0.71) and 0.60 (*95% CI* 0.53–0.67), respectively. Postbronchodilator FEV_1_% predicted at 71.40% exhibited the optimal sensitivity (51.39%) and specificity (79.81%), and peripheral blood eosinophil counts at 0.60 × 10^9^/L exhibited the optimal sensitivity (41.67%) and specificity (77.88%) for predicting the diagnosis of bronchiectasis (Table 4).

**Table 4:** Differential diagnostic values of postbronchodilator FEV_1_% predicted, peripheral blood eosinophil counts and combined model in detecting bronchiectasis in asthma-CRS overlap patients

Items	Cutoff value	Sensitivity (%)	Specificity (%)	Youden index
postbronchodilator FEV_1_% predicted, %	71.40	51.39	79.81	0.31
peripheral blood eosinophil counts, × 10^9^/L	0.60	41.67	77.88	0.20
combined model	0.33	72.22	70.19	0.42

*FEV*_*1*_ forced expiratory volume in 1 s

The AUC of the combined model with postbronchodilator FEV_1_% predicted ≤ 71.40%, peripheral blood eosinophil counts > 0.60 × 10^9^/L, the presence of NPs, and ≥ 1 severe exacerbation of asthma in the last 12 months was 0.75 (*95% CI* 0.68–0.81). The AUC of the combined model predicted better diagnostic accuracy than NPs, ≥ 1 severe exacerbation of asthma in the last 12 months, postbronchodilator FEV_1_% predicted or peripheral blood eosinophil counts (*P* < 0.001).

### Extent and severity of bronchiectasis predict a more severe asthma-CRS subset

**Fig. 4:**
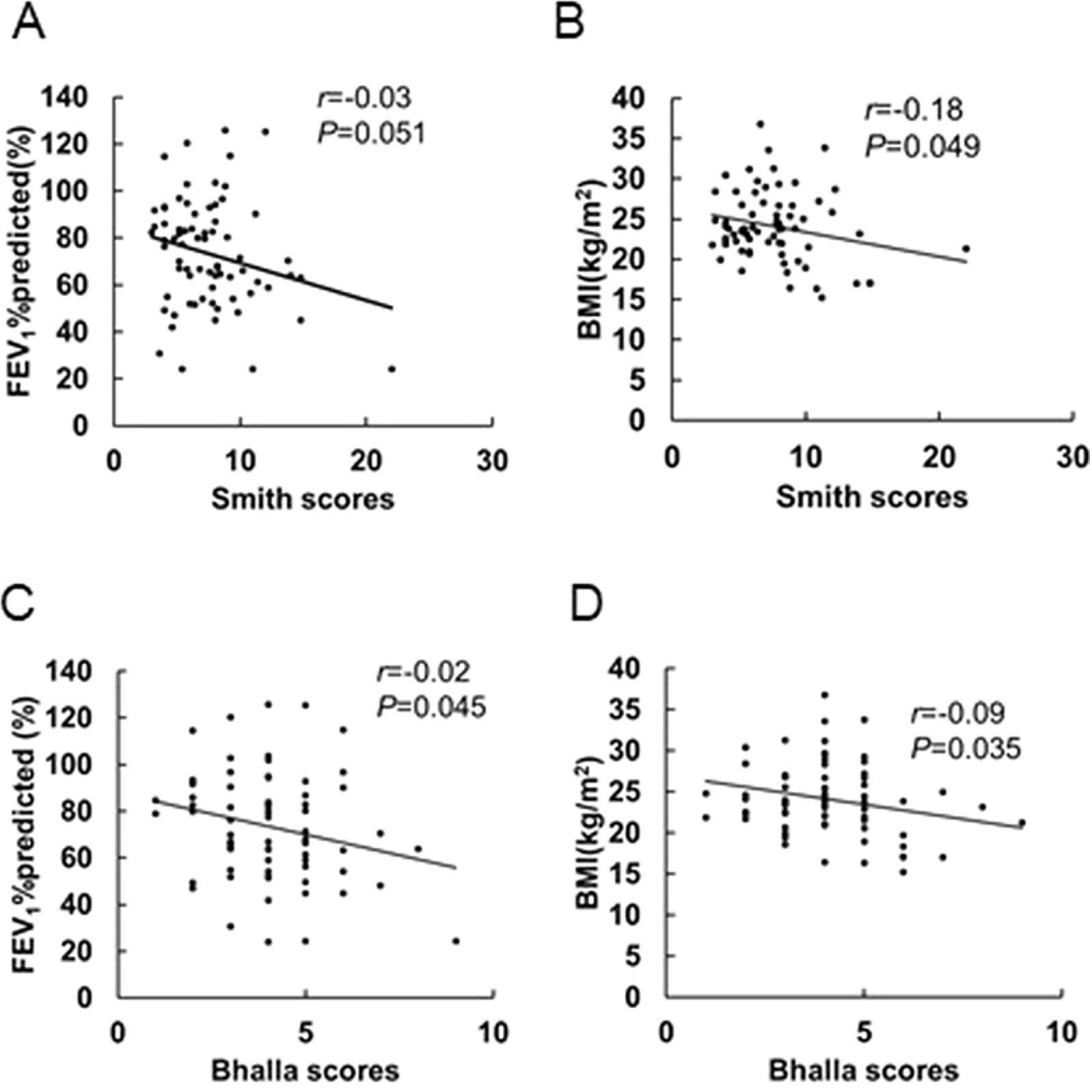
Correlation analysis in the bronchiectasis group. **A** Scatter plot between Smith scores and postbronchodilator FEV_1_% predicted. **B** Scatter plot between Smith scores and BMI. **C** Scatter plot between Bhalla scores and postbronchodilator FEV_1_% predicted. **D** Scatter plot between Bhalla scores and BMI

Univariate analyses showed that Smith scores were associated with lower BMI (*P* = 0.049). Although not significant, Smith scores also correlated with FEV_1_% predicted (*P* = 0.051), NP occurrence (*P* = 0.088) and ≥ 1 pneumonia in the last 12 months (*P* = 0.091) (Table 5, Fig. 4A, B). On the other hand, Bhalla scores were significantly associated with lower BMI (*P* = 0.035), higher FeNO (*P* = 0.044), lower FEV_1_% predicted (*P* = 0.045) and a higher occurrence ratio of NPs (*P* = 0.026). Bhalla scores also correlated with higher LM scores (*P* = 0.060) and positive smoking status (*P* = 0.050), although these results were not significant (Table 6, Fig. 4C, D).

**Table 5:** Univariate analyses of correlated factors for Smith scores in bronchiectasis patients

Variables	Smith Score		
	*r*	*95% CI*	*P* value
Male sex	0.04	− 1.53 to 1.62	0.955
Age	0.01	− 0.05 to 0.07	0.760
BMI	− 0.18	− 0.35 to 0.00	0.049
Positive smoking status^a^	0.90	− 0.75 to 2.56	0.280
Smoking index^b^	0.19	− 0.23 to 0.59	0.367
Duration of asthma	0.01	− 0.04 to 0.06	0.620
NPs	1.35	− 0.21 to 2.90	0.088
Prior sinus surgery	0.22	− 1.60 to 2.04	0.811
Allergic rhinitis	− 0.37	− 2.06 to 1.31	0.662
Atopic dermatitis	− 1.75	− 5.67 to 2.16	0.375
Gastroesophageal reflux disease	− 0.12	− 3.21 to 2.98	0.941
ICS dose (fluticasone equivalent)	0.00	0.00 to 0.00	0.812
Severe asthma	0.82	− 1.10 to 2.75	0.397
≥ 1 severe exacerbation of asthma in the last 12 months	1.14	− 0.43 to 2.70	0.153
≥ 1 pneumonia in the last 12 months	1.40	− 0.23 to 3.04	0.091
Peripheral blood eosinophil count	0.86	− 0.33 to 2.05	0.153
FeNO	0.01	− 0.01 to 0.03	0.274
Total IgE	0.00	0.00 to 0.00	0.476
Atopy	− 0.98	− 2.54 to 0.58	0.214
postbronchodilator FEV_1_% predicted	− 0.03	− 0.07 to 0.00	0.051
LM scores	0.10	− 0.02 to 0.22	0.117

*BMI* body mass index, *NPs* nasal polyps, *FEV*_*1*_ forced expiratory volume in 1 s, *ICS* inhaled corticosteroid, *FeNO* fractional exhaled nitric oxide, *IgE* immunoglobulin E, *LM* Lund-Mackay

^a^Positive smoking status included ex- and current-smokers

^b^Smoking history in patients with positive smoking status

**Table 6:** Univariate analyses of correlated factors for Bhalla scores in bronchiectasis patients

Variables	Bhalla score		
	*r*	*95% CI*	*P* value
Male sex	0.19	− 0.54 to 0.92	0.602
Age	0.01	− 0.01 to 0.04	0.368
BMI	− 0.09	− 0.17 to − 0.01	0.035
Positive smoking status^a^	0.75	0.00 to 1.50	0.050
Smoking index^b^	0.08	− 0.12 to 0.27	0.429
Duration of asthma	− 0.00	− 0.03 to 0.02	0.811
NPs	0.81	0.10 to 1.51	0.026
Prior sinus surgery	0.02	− 0.82 to 0.86	0.965
Allergic rhinitis	0.06	− 0.72 to 0.84	0.876
Atopic dermatitis	− 1.09	− 2.88 to 0.71	0.232
Gastroesophageal reflux disease	− 0.69	− 2.11 to 0.73	0.335
ICS dose (fluticasone equivalent)	0.00	− 0.00 to 0.00	0.468
Severe asthma	0.62	− 0.26 to 1.50	0.164
≥ 1 severe exacerbation of asthma in the last 12 months	0.27	− 0.46 to 1.00	0.469
≥ 1 pneumonia in the last 12 months	0.50	− 0.26 to 1.26	0.194
Peripheral blood eosinophil counts	0.19	− 0.36 to 0.74	0.493
FeNO	0.01	0.00 to 0.02	0.044
Total IgE	0.00	0.00 to 0.00	0.468
Atopy	− 0.30	− 1.03 to 0.42	0.408
Postbronchodilator FEV_1_% predicted	− 0.02	− 0.03 to 0.00	0.045
LM scores	0.05	0.00 to 0.11	0.060

*BMI* body mass index, *NPs* nasal polyps, *FEV*_*1*_ forced expiratory volume in 1 s, *ICS* inhaled corticosteroid, *FeNO* fractional exhaled nitric oxide, *IgE* immunoglobulin E, *LM* Lund-Mackay

^a^Positive smoking status included ex- and current-smokers

^b^Smoking history in patients with positive smoking status

Multivariate linear regression showed that the Smith scores were positively correlated with NP occurrence (*β coefficients*: 1.74; *95% CI* 0.250–3.24) and negatively correlated with postbronchodilator FEV_1_% predicted (*β coefficients*: − 0.04; *95% CI* − 0.07 to − 0.01) and BMI (*β coefficients*: − 0.18; *95% CI* − 0.34 to − 0.01). Bhalla scores were positively correlated with NPs (*β coefficients*: 1.11; *95% CI* 0.45 to 1.77) and positive smoking status (*β coefficients*: 0.73; *95% CI* 0.03–1.44) and negatively correlated with postbronchodilator FEV_1_% predicted (*β coefficients*: − 0.02; *95% CI* − 0.03 to − 0.00) and BMI (*β coefficients*: − 0.08; *95% CI* − 0.16 to − 0.00) (Table 7).

**Table 7:** Multivariate linear regression analyses for Smith scores and Bhalla scores

Variables	Smith score: multivariate linear regression		
	*β* coefficients	*95% CI*	*P* value
NPs	1.74	0.25 to 3.24	0.023
Postbronchodilator FEV_1_% predicted	− 0.04	− 0.07 to − 0.01	0.019
BMI	− 0.18	− 0.34 to − 0.01	0.041

Variables	Bhalla score: multivariate linear regression		
	*β* coefficients	*95% CI*	*P* value
NPs	1.11	0.45 to 1.77	0.001
Postbronchodilator FEV_1_% predicted	− 0.02	− 0.03 to − 0.00	0.029
BMI	− 0.08	− 0.16 to − 0.01	0.032
Positive smoking status^a^	0.73	0.03 to 1.44	0.042

*BMI* body mass index, *FEV*_*1*_ forced expiratory volume in 1 s, *NPs* nasal polyps

^a^Positive smoking status included ex- or current-smokers

## Discussion

As a typical “united airways” disease, the asthma-CRS overlap has recently drawn the attention of respiratory physicians, otolaryngologists and allergists [3, 18, 27]. Bronchiectasis is a heterogeneous disease related to a variety of diseases. Whether bronchiectasis exists and correlates with asthma-CRS patients has not been fully elucidated. In this study, we summarized the prevalence of bronchiectasis in asthma-CRS patients, and furthermore, we analyzed the characteristics of a novel disease subset, bronchiectasis overlapping with asthma-CRS in the united airway.

Bronchiectasis is a chronic bronchial disorder characterized by permanent and irreversible destruction and dilatation of the bronchial wall leading to chronic airway inflammation and bacterial colonization [5, 8, 21]. When used to describe a disease, bronchiectasis includes a heterogeneous group of disorders that differ significantly in terms of etiological, clinical, radiological, functional and microbial features. Previous studies have revealed overlaps between bronchiectasis and chronic upper and lower airway diseases. Bronchiectasis has been reported in 3–47% of patients initially diagnosed with asthma [6, 10] and in approximately 5.5% of patients with CRS [1]. In our cohort, we found that 40.9% of asthma-CRS patients could also be codiagnosed with bronchiectasis (Table 1), indicating that bronchiectasis was popular in this group of patients.

Generally, bronchiectasis is primarily mediated by neutrophilic inflammation, which is closely linked to persistent bacterial infection [5]. In contrast, inflammatory cells are dominantly eosinophils in the pathogenesis of asthma and CRS [3, 18, 28]. We showed that the existence of bronchiectasis in asthma-CRS patients was associated with elevated peripheral blood eosinophil counts and IgE levels, indicating that the nature of the inflammatory pattern in bronchiectasis patients with asthma-CRS overlap is eosinophilic rather than neutrophilic. In line with our studies, previous studies also showed that elevated eosinophilic inflammation may be correlated with more severe remodeling in the large- to medium-sized airway, bronchial wall thickening, mucus plugging, and bronchiectasis in asthma patients [28, 29]. Steroid-dependent asthma is predominately mediated by eosinophilic airway inflammation and is more likely to be associated with bronchiectasis [30], indicating that eosinophilic airway inflammation is related to bronchiectasis formation in asthma patients. Similarly, recent data also found that peripheral eosinophil counts were elevated in patients with bronchiectasis and CRS [13]. Bronchiectasis may be induced by eosinophil infiltration and eosinophil-derived cationic proteins, lipid mediators, cytokines, chemokines, and growth factors [5]. Hence, we believe that ICS and eosinophilic targeted therapy (such as IL-5 antibody) are beneficial for the distinct disease subset with bronchiectasis-asthma-CRS overlapping in the same airway. However, in contrast with our study, Padilla—Galo et al. [15] found that bronchiectasis was related to lower levels of FeNO, which indicates a neutrophil infiltration pattern. Notably, the OR for FeNO in their results was 0.98, close to 1, indicating that the correlation was not strong. In addition, it is plausible that bronchiectasis in asthma-CRS patients correlates with both “eosinophilic-high” and “neutrophilic-high” inflammation patterns. Thus, precise, individualized treatment (anti-eosinophilic, anti-neutrophilic, or both) based on the underlying heterogeneous airway/circulating inflammation pattern for bronchiectasis-asthma-CRS overlapping patients should be further studied. Moreover, one post hoc analysis of a randomized clinical trial showed that the presence of eosinophils can occur in bronchiectasis patients even without asthma [31]. Most likely, bronchiectasis itself can be divided into "eosinophilic-high" and "neutrophilic-high" inflammation patterns with different mechanisms, clinical characteristics and therapy strategies.

In the current study, we showed that overlapping with bronchiectasis in asthma-CRS patients had a higher proportion of ≥ 1 severe exacerbation of asthma in the last 12 months and a lower predicted FEV_1_%. This outcome indicated that patients with both disorders in the same airway generally showed a more severe disease subset and worse prognosis. In line with our studies, previous studies also showed that bronchiectasis was more likely to exist in severe asthma with frequent exacerbations [6, 11, 32–34]. Asthma overlapping with bronchiectasis represents a distinct disease subset with a poorer prognosis in terms of asthma exacerbations and resistance to current asthma treatment. Therefore, we suggest that although the exact causal relationship between radiological bronchiectasis and asthma-CRS is currently unclear, it is plausible to hypothesize that the predisposing airway infiltration of eosinophils in asthma-CRS patients may induce persistent airway inflammation, airway remodeling and mucus plug removal impairment, which further leads to the development of bronchiectasis. Thus, bronchiectasis can impair lung function and induce frequent exacerbations of asthma and eventually promote the production of a specific disease subset associated with worse prognosis. However, how bronchiectasis and asthma-CRS overlap arise (i.e., a causal connection or a chance association) should be further investigated.

Generally, CRS can be divided into CRS with NPs (CRSwNPs) and CRS without NPs (CRSsNPs) based on the presence or absence of NPs. In contrast with CRSsNPs, CRSwNPs is mostly dominated by eosinophilic inflammation [3]. Our study showed that the presence and severity of radiological bronchiectasis was associated with NP occurrence (Tables 3 and 7). In line with our study, Canonica et al*.* also demonstrated that bronchiectasis was more common in patients with CRSwNPs than in those with CRSsNPs [2]. In view of this finding, the overlap of asthma, CRSwNPs, and bronchiectasis in the same airway may represent a distinct disease subset with eosinophilic airway inflammation instead of neutrophils. Thus, targeted therapy for CRSwNP patients, e.g., ICS, anti-allergic drugs, and polypectomy, may also be beneficial for patients with overlapping radiological bronchiectasis. However, the definite causal relationship between CRSwNPs and radiological bronchiectasis in the context of unified airway eosinophilic inflammation is currently unclear. Large cohort, long-term, and follow-up studies using patients with CRSwNPs and radiological bronchiectasis alone or overlapping are needed to resolve this open question.

Comprising all of the above, we established a combined model to predict the presence of bronchiectasis from asthma-CRS patients, with postbronchodilator FEV_1_% predicted ≤ 71.40%, peripheral blood eosinophil counts > 0.60 × 10^9^/L, the presence of NPs, and ≥ 1 severe exacerbation of asthma in the last 12 months. It is recommended to perform chest HRCT to monitor and intervene in bronchiectasis early, especially for asthma-CRS patients with these characteristics.

Our findings showed that the BMI of patients with asthma-CRS bronchiectasis decreased as the degree of bronchiectasis deteriorated. Similarly, previous studies also indicated that bronchiectasis can lead to malnutrition with lower BMI in patients with asthma [35]. Bronchiectasis patients with a lower BMI were prone to develop more acute exacerbations, worse pulmonary function and higher risk of death because of amplified systemic inflammation and chronic bacterial colonization [35, 36]. Taken together, our results suggest that the severity of bronchiectasis in asthma-CRS patients predicts a poor nutritional status and quality of life and should be surveilled and treated.

Several previous studies have described the adverse effects of smoking on asthma and CRS [37, 38], and similarly, smoking was an independent risk factor for the severity and prognosis of bronchiectasis [39]. Our study also demonstrated that there was a positive correlation between the severity of bronchiectasis and positive smoking status. Thus, smoking cessation in asthma-CRS patients is strongly advised, especially in patients with overlapping bronchiectasis.

## Limitations

There were several limitations in this study. First, this is a clinical study. Further study on the molecular mechanism may provide a basis to explore the pathogenesis of the asthma-CRS-bronchiectasis subset. Second, this study involved asthmatic patients with stable status, and further research focusing on inflammatory characteristics during the exacerbation of asthma may more fully evaluate the relationship of bronchiectasis and asthma-CRS with different stages. Third, due to a lack of microbiological information, the role of infection in the presence and development of bronchiectasis in asthma-CRS needs further investigation. Last, as this is a single-center study with a limited sample size, external validation, such as a multicenter study or study of other races, needs to be carried out to verify the conclusion.

## Conclusions

The coexistence of radiographic bronchiectasis in asthma-CRS patients is common and predicts a distinct disease subset with more severe eosinophilic airway inflammation, more serious asthma and CRS, and lower quality of life. Subgroups of asthma-CRS patients with NPs, more severely impaired lung function, higher circulating levels of eosinophils, and more frequent acute asthma attacks should receive HRCT examination for earlier diagnosis and treatment of bronchiectasis.

## Data Availability

The datasets used and analyzed for this study are available from the corresponding author on reasonable request.

## Abbreviations

CRS: Chronic rhinosinusitis
LM: Lund-Mackay
HRCT: High-resolution CT
NPs: Nasal polyps
GINA: Global Initiative for Asthma
FEV_1_: Forced expiratory volume in one second
COPD: Chronic obstructive pulmonary disease
IgE: Immunoglobulin E
FeNO: Fraction of exhaled nitric oxide
FVC: Forced vital capacity
ROC: Receiver operating characteristic
AUC: Area under the curve
